# Supplementary material for: Content-rich biological network constructed by mining PubMed abstracts
Source: BMC Bioinformatics. 2004 Oct 8;5:147. doi: 10.1186/1471-2105-5-147 (PMC528731; doi:10.1186/1471-2105-5-147)
Supplement: Additional File 2 — The original results of the above study (non-essential files are deleted to keep the file size under the limit set by BMC bioinformatics). [file 1471-2105-5-147-S2.bz2 › chilibotAdditionalFile2/dip30/500ID8041735E10665/html/TGFB2_TGFBR3.html]

 


 **TGFB2** and **TGFBR3** 
  
Found 27 abstracts in PubMed, retrieved 27.  
 

 What does Google say? 
 PDF only 
| .edu only 

---

**Interactive relationship** (e.g. stimulation, inhibition, etc)

**Neutral relationship**- Ligand competition assays indicate that the relative affinities of recombinant soluble betaglycan  [ **TGFBR3** ]  for the TGF beta isoforms are TGF beta2  [ **TGFB2** ] >TGF beta3>TGF beta1.  Ref: 11256966 Biochem J, 2001
- The anti TGF beta potency of recombinant soluble betaglycan  [ **TGFBR3** ]  in vitro is 10 fold higher for TGF beta2  [ **TGFB2** ]  than for TGF beta1.  Ref: 11256966 Biochem J, 2001
- Compared with a commercial pan specific anti TGF beta neutralizing antibody, recombinant soluble betaglycan  [ **TGFBR3** ]  is more potent against TGF beta2  [ **TGFB2** ]  and similar against TGF beta1.  Ref: 11256966 Biochem J, 2001
- These experiments demonstrate that the betaglycan  [ **TGFBR3** ]  component which exhibits a higher affinity for TGF beta 2  [ **TGFB2** ]  than for TGF beta 1, that we had observed previously on term placental membranes, is actually present on trophoblast cells.  Ref: 1310325 J Cell Physiol, 1992
- The results from affinity labelling saturation and competition experiments with TGF beta 1 and TGF beta 2  [ **TGFB2** ]  suggest the existence of two distinct subtypes of betaglycan  [ **TGFBR3** ]  one subtype has a lower capacity and higher affinity, binds both TGF beta 1 and TGF beta 2  [ **TGFB2** ] , yet has a preferential affinity for TGF beta 2  [ **TGFB2** ] .  Ref: 1310325 J Cell Physiol, 1992
- The 38 kDa TGF beta binding component, an N linked glycoprotein, exhibits a higher affinity for TGF beta 2  [ **TGFB2** ]  than for beta 1 that is strikingly similar to that of the type III betaglycan  [ **TGFBR3** ]  receptor.  Ref: 1333844 Mol Biol Cell, 1992
- Purified betaglycan  [ **TGFBR3** ]  has properties similar to betaglycan  [ **TGFBR3** ]  affinity labeled in intact cells it binds TGF beta 1 and TGF beta 2  [ **TGFB2** ]  with KD approximately 0.2 nM, contains heparan sulfate and chondroitin sulfate glycosaminoglycan GAG chains and N linked glycans attached to a 110 kDa core protein, and can spontaneously associate with phosphatidylcholine liposomes.  Ref: 1744125 J Biol Chem, 1991
- Affinity labeling saturation and competition studies indicated that the type III betaglycan  [ **TGFBR3** ]  component appears to have a 7 fold higher capacity for TGF beta 1 than for beta 2 yet exhibits a 5 to 10 fold higher affinity for TGF beta 2  [ **TGFB2** ]  than for beta 1.  Ref: 1333844 Mol Biol Cell, 1992
- Forced expression of betaglycan  [ **TGFBR3** ]  in CC myoblasts increases their responsiveness to TGF beta2  [ **TGFB2** ] , suggesting that it performs a TGF beta presentation function in this cell lineage.  Ref: 12399463 J Biol Chem, 2003
- In fact, recombinant soluble betaglycan  [ **TGFBR3** ]  acts as potent inhibitor of TGF beta binding to membrane receptors and blocks TGF beta action, this effect being particularly pronounced with the TGF beta 2  [ **TGFB2** ]  isoform.  Ref: 8106553 J Cell Biol, 1994

**Non-interactive relationship** (e.g. studied together, co-existance, homology, etc.)

- Subtypes of betaglycan  [ **TGFBR3** ]  and of type I and type II transforming growth factor beta TGF beta receptors with different affinities for TGF beta 1 and TGF beta 2  [ **TGFB2** ]  are exhibited by human placental trophoblast cells.  Ref: 1310325 J Cell Physiol, 1992
- In addition to receptors I and II and betaglycan  [ **TGFBR3** ] , some cells express a newly identified set of membrane proteins that specifically bind either TGF beta 1 or TGF beta 2  [ **TGFB2** ] .  Ref: 1322148 Mol Reprod Dev, 1992
- Betaglycan  [ **TGFBR3** ]  binds TGF beta 1 and TGF beta 2  [ **TGFB2** ]  with similar affinity and this binding is to the core proteins, not the glycosaminoglycan side chains.  Ref: 1964683 J Cell Sci Suppl, 1990
- Following abrogation of betaglycan  [ **TGFBR3** ]  expression with antisense oligodeoxynucleotide, embryonic lungs were relatively insensitive to TGF beta TGF beta2  [ **TGFB2** ]  0.5 ng ml and TGF beta1 20 ng ml, respectively, down regulated lung morphogenesis by 38 and 34% in control cultures.  Ref: 9739109 Mech Dev, 1998
- We therefore studied the expression of UPA, UPAR, IGF2R, ALK5, TGFBR2,  **TGFBR3** , ENG, ALK1, TGFB1,  **TGFB2** , and TGFB3 in a series of 14 pancreatic carcinoma cell lines.  Ref: 11408925 Int J Oncol, 2001
- Motoneurons were shown to synthesize the three proteins involved in transforming growth factor beta 2  [ **TGFB2** ]  signalling types I and II transforming growth factor beta receptor and betaglycan  [ **TGFBR3** ]  and to transport them anterogradely, where they were inserted into the axonal membrane and nerve terminal.  Ref: 10842018 Neuroscience, 2000
- Affinity labeling studies with 125I TGF beta 1 and 125I TGF beta 2  [ **TGFB2** ]  and the chemical cross linking agent bis sulfosuccinimidyl suberate BS3 revealed a predominant type III betaglycan  [ **TGFBR3** ]  receptor, a low level of apparently heterogeneous type I and II receptors and an additional novel 38 kDa TGF beta binding glycoprotein that was present both under reducing and nonreducing conditions on sodium dodecyl sulfate polyacrylamide gel electrophoresis SDS PAGE.  Ref: 1333844 Mol Biol Cell, 1992
